# Whole body vibration training during allogeneic hematopoietic cell transplantation—the effects on patients’ physical capacity

**DOI:** 10.1007/s00277-020-03921-x

**Published:** 2020-01-23

**Authors:** Antonia Pahl, Anja Wehrle, Sarah Kneis, Albert Gollhofer, Hartmut Bertz

**Affiliations:** 1 Department of Medicine I, Medical Center-University of Freiburg, Hugstetterstr. 55, 79106 Freiburg, Germany; 2 Institute for Exercise and Occupational Medicine, Medical Center-University of Freiburg, Hugstetterstr. 55, 79106 Freiburg, Germany; 3 Department of Sport and Sport Science, University of Freiburg, Schwarzwaldstraße 175, 79117 Freiburg, Germany

**Keywords:** Stem cell transplantation, Resistance exercise, Exercise therapy, Leukemia, Side effects, Galileo®

## Abstract

Patients undergoing allogeneic hematopoietic cell transplantation (alloHCT) experience a considerable decline in physical and psycho-social capacity. Since whole body vibration (WBV) is known to efficiently stimulate the neuromuscular system and enhance cardiorespiratory fitness and muscle strength in frail individuals, we hypothesized that WBV would maintain various physical and psychological capacities in patients during alloHCT. Seventy-one patients were randomly allocated to either an intervention group (IG) doing WBV or an active control group (CG) doing mobilization exercises five times per week. We determined peak oxygen consumption (VO_2peak_) and maximum power, maximum strength, functional performance, body composition, quality of life (QoL), and fatigue. Tests were carried out before conditioning therapy, at hospital discharge and at day ± 180 (follow-up). As 18 patients did not participate in post-intervention assessment and follow-up data from 9 patients was not collectible, per-protocol (PP) analysis of 44 patients is presented. During hospitalization, WBV maintained maximum strength, height, and power output during jumping, as well as reported QoL, physical functioning, and fatigue level compared with mobilization. At follow-up, relative VO_2peak_ (*p* = 0.035) and maximum power (*p* = 0.011), time and power performing chair-rising test (*p* = 0.022; *p* = 0.009), and reported physical functioning (p = 0.035) significantly increased in the IG, while fatigue decreased (*p* = 0.005). CG’s body cell mass and phase angle had significantly decreased at follow-up (*p* = 0.002; *p* = 0.004). Thus, WBV might maintain maximum strength, functional performance, QoL, and fatigue during alloHCT, while cardiorespiratory fitness might benefit from accelerated recovery afterwards.

**Electronic supplementary material:**

The online version of this article (10.1007/s00277-020-03921-x) contains supplementary material, which is available to authorized users.

## Background

Allogeneic hematopoietic cell transplantation (alloHCT) is usually associated with hospitalization lasting at least 4 weeks. The treatment itself, therapy-related side effects, and bed rest and physical inactivity lead to a general decline in physical performance, maximum oxygen consumption, muscular performance in particular, and quality of life (QoL) as a consequence of all impairments [1–5]. Poor maximum oxygen consumption and peak exercise capacity are associated with a higher risk of cardiovascular diseases and all-cause mortality [6–9]. Furthermore, muscle mass and strength loss are inter alia associated with higher risk of falls and mortality in cancer patients [10–12]. These impairments thus restrict patients’ autonomy, QoL, and ultimately, their overall survival [13, 14]. To counteract such declines, several studies investigated the effects of exercises during therapy [15–18]. There is ample evidence that physical exercise in form of aerobic or multimodal exercise programs (including moderate resistance exercises) is feasible and can positively influence patients’ functional performance even during alloHCT [19–22]. Nonetheless, it remains unclear how cardiopulmonary fitness and muscle strength can both be maintained most efficiently. Considering that muscular capacity is one predictor for maximum oxygen consumption [23], focusing on muscle-preserving exercises might be particularly relevant for alloHCT patients. Cunningham et al. [24] showed that resistance training can counteract protein degradation initiated by bed rest and medical treatment and may therefore maintain muscle mass during alloHCT. However, engaging in conventional resistance training during alloHCT is restricted according to current recommendations. Thus, patients’ blood values, i.e., platelets counts, and well-being limit the intensity and volume of resistance training [25, 26]. Therefore, whole body vibration (WBV) presents a gentler resistance training method, as it does not additionally exacerbate cardiovascular stress while exercising [27], and reveals similar EMG activity as resistance exercises with external loads [28]. WBV is applied through a vibration platform upon which the subjects stand; it produces alternating sinusoidal movements of the lower body [29]. Thus, WBV induces involuntary, frequency-dependent, repeating eccentric, and concentric muscle contractions in the legs culminating in a tonic vibration reflex that enhances the recruitment of motor units during static or dynamic exercises [30, 31]. Consequently, WBV, similar to low-intensity electrical muscle stimulation [32], enables high neuromuscular activity that can improve functional performance as well as cardiorespiratory fitness [33–35], may increase muscle strength [36], and is known to prevent muscle-mass loss during bed rest in healthy individuals [4, 37]. In patients with chronic diseases like neurological, musculoskeletal, or metabolic disorders, WBV has proven to improve muscle strength, mobility, and balance [38]. In heart transplant recipients, Crevenna et al. [39] reported heart rates, blood pressures, and lactate concentrations after WBV that were similarly increased after aerobic endurance exercises. Thus, provided patients have no acute infection, restrictive orthopedic disease in the lower body, or untreated cardiovascular illness, no adverse events are likely [40]. We already proved its feasibility for hospitalized patients undergoing high-dose chemotherapy in a previous investigation [41]. Therefore, we hypothesized that WBV would maintain patients’ cardiorespiratory fitness as a reliable representative of physical capacity and trigger further adaptations in the neuromuscular system, body composition, and QoL. Consequently, we implemented a stratified randomized controlled trial to assess the effects of WBV on patients undergoing alloHCT.

## Methods

### Study design and patients

Between June 2016 and October 2017, all consecutive eligible patients were recruited at the Department of Medicine I, University Medical Center Freiburg, Germany, at the day of hospital admission for alloHCT. Seventy-one patients were randomly allocated 1:1 to two parallel groups: an intervention group (IG) or an active control group (CG). Stratified randomization was based on patients’ sex and conditioning chemotherapy protocol. Randomization in blocks of 10 was based on a computer-assisted pseudo-random number generator (Research Randomizer, Version 4.0). Allocation was implemented by sequentially numbered, sealed, opaque envelopes. After obtaining patient’s consent, the researcher opened the next consecutively numbered envelope. Inclusion criteria were patients scheduled for alloHCT, over the age of 18 years, able to perform a cardiopulmonary exercise test, and written informed consent. Exclusion criteria were unstable bone metastasis, endoprosthesis of knee or hip, epilepsy, pacemaker, and severe cardiovascular diseases according to other studies [42, 43]. Furthermore, patients’ blood values had to fulfill safety criteria for the testing of maximum capacity on the respective day (platelets count ≥ 20.000/μl and hemoglobin ≥ 8 g/dl blood) [44, 45]. Parameters were assessed before conditioning therapy (baseline, T0), at hospital discharge (T1), and at follow-up about day ± 180 post transplantation (T2). Table 1 summarizes patients’ clinical information. The study was approved by the Ethics Committee of the University of Freiburg and conducted according to the Declaration of Helsinki (German Register of Clinical Trials No.: DRKS00009918).

**Table 1:** Patients’ characteristics

	IG *n* = 18	CG *n* = 26
Age (years)^#^	55 (50–63)	56 (32–63)
Sex (*N*)		
Male:female	11:7	19:7
BMI (kg/m^2^)^#^	26.1 (20.1–28.2)	26 (22.4–28.1)
Diagnosis (*n*)		
AML	13	13
ALL	1	2
CLL	0	1
CMML	1	0
MDS	1	2
lymphoma	1	3
MM	0	1
Myelofibrosis	1	0
Septic granulomatosis	0	1
Common variable immunodeficiency	0	1
SAA	0	2
Remission at alloHCT (*n*)		
SD	0	1
PD	6	7
CR	8	9
PR	1	1
Recurrence	0	1
PIF	1	3
Progression	0	2
Untreated	1	1
NMD	0	1
N.a.	1	0
HCT-CI score (score)^#^	2 (1–3)	2 (0–3)
EBMT score (score)^#^	5 (3.5–5)	5 (4–5)
Karnofsky performance index (%)^#^	90 (85–90)	90 (90–95)
Cycles of chemotherapy (*n*)^#^	2 (1–4)	1 (0–9)
Hospitalization during alloHCT (days)^#^	38 (35–43.5)	41 (37–44)

^#^Median (range)

*BMI:* body mass index; *AML*: acute myeloid leukemia; *ALL*: acute lymphocytic leukemia; *CLL*: chronic lymphatic leukemia; *CMML*: chronic myelo-monocytic leukemia; *MDS*: myodysplatic syndrom; *MM*: multiple myeloma; *SAA*: severe aplastic anemia; *SD*: stable disease; *PD*: progress disease; *CR*: complete remission; *PR*: partial remission; *PIF*: persistent induction failure; *NMD*: non-malignant disease; *HCT-CI*: hematopoietic cell transplantation-comorbidity index; *EBMT*: European Group for Blood and Marrow Transplantation

### Intervention

Both groups’ one-on-one training sessions took place in the patient’s room. Both groups’ intervention protocol prescribed daily exercising on weekdays for approximately 20 min if justified by patients’ well-being and blood values that had to fulfill safety criteria for exercising: platelets count ≥ 10.000/μl and hemoglobin ≥ 7.5 g/dl without dizziness.

The IG performed WBV training of the legs standing on the side-alternating Galileo® Basic vibration platform (Novotec Medical GmbH, Pforzheim, Germany). CG performed mobilization of the spine and stretching of the whole body sitting or lying in bed or standing in front of it. For detailed information, see Supplementary file 1 and 2.

### Outcome measures

In the following, a short description of outcome measures is presented. For detailed information, see Supplementary file 1.

#### Primary endpoint

##### Peak oxygen consumption (VO2peak)

VO2peak (l/min) was measured during maximum cardiopulmonary exercise test (CPET).

#### Secondary endpoints

##### Cardiorespiratory fitness

Cardiorespiratory fitness parameters were also measured during cardiopulmonary exercise test (CPET) in consideration of patients’ body weight: peak oxygen consumption (ml/min/kg) and maximum power output (W/kg).

##### Strength capacity

We determined maximum voluntary strength of the knee extensors and flexors (Nm) and muscular endurance of those muscles (%) via isokinetic measurement (CONT-TREX MJ, CMV, Duebendorf, Switzerland).

##### Functional performance

All functional performance measurements were taken on a force plate (Leonardo Mechanograph® GRFP, Novotec Medical GmbH, Pforzheim, Germany) that determined dynamic ground reaction forces in their local and temporal progress.

Two common functional tasks were performed: the chair-rising test and maximum counter-movement jump. We calculated the duration performing one repetition (s) and power output while getting up (W/kg). For the counter-movement jump, we calculated maximum power output during take-off per kilogram body weight (W/kg) and jumping height (cm). Both tests evaluate leg muscle power.

##### Body composition

To determine body fat (%; kg) and fat-free mass (%), an air displacement plethysmography system (Bod Pod Body, Composition System, Life Measurement, INC) as well as bioelectrical impedance analysis (BIA, Nutriguard-S, Data Input, Pöcking, Germany) was used. Via bioelectrical impedance analysis, also body cell mass (kg) and phase angle (°) were determined.

##### Quality of life and fatigue

The EORTC QLQ-C30-questionnaire (European Organization for Research and Treatment of Cancer Quality of Life) was used to assess QoL in general, and for transplantation-associated physical symptoms, the EORTC-QLQ-HDC29 was also applied.

To measure fatigue, we used the Multidimensional Fatigue Inventory questionnaire.

##### Physical activity

To assess patients’ physical activity before, since general cancer therapy, and after alloHCT, we modified the Freiburg questionnaire on physical activity (FFKA modified) according to the time interval of interest. For analysis, we grouped three categories when physical activity can be carried out: daily routine, leisure time, and sports.

### Sample size and statistics

The primary endpoint VO_2peak_ measured at hospital discharge was analyzed in a linear regression model with treatment allocation, baseline VO_2peak_, gender, and age as independent variables. As the assumption of normal distribution (Shapiro-Wilk-test) was not satisfied and because of a 38% dropout rate, all variables and endpoints were included in non-parametric per-protocol analysis. Differences in all variables between both groups and differences in groups’ delta (T1-T0 and T2-T0) were assessed by Mann-Whitney *U* test. Intragroup differences over time were computed by Wilcoxon signed-rank test. The level of significance was set to *p* < 0.05. Group data are presented as median and 95% confidence interval (95% CI). The point estimate and 95% confidence interval of the Hodges-Lehmann’s median differences for paired groups were used to estimate the treatment effect. All statistical analyses were conducted using the IBM SPSS Version 22 software (SPSS Inc., Chicago, Illinois, USA).

## Results

**Fig. 1:**
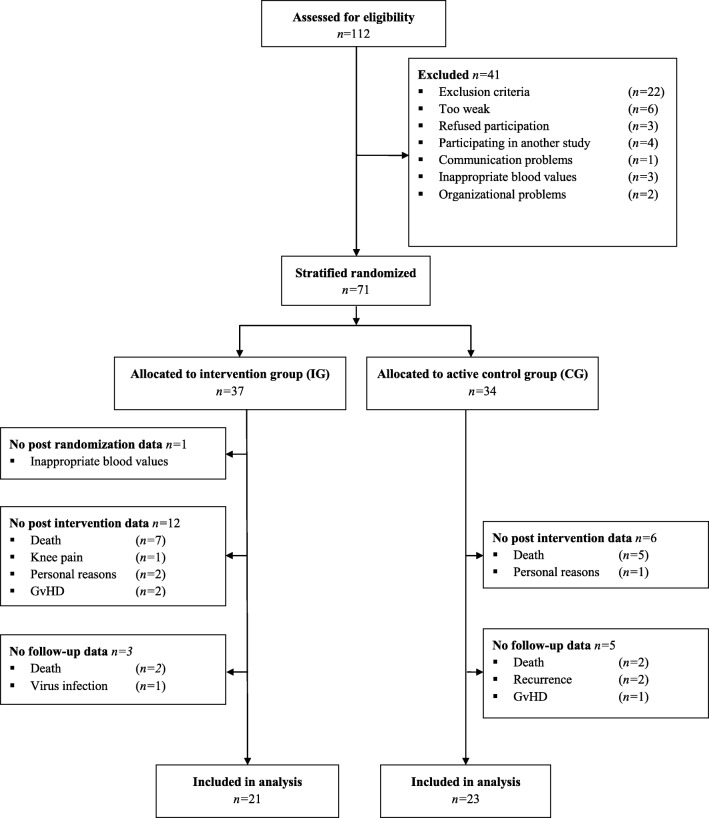
Flow diagram of patient recruitment

As 18 patients did not participate in post-intervention assessment and follow-up data from 9 patients was not collectible (Fig. 1), we present a per-protocol (PP) analysis of 44 patients who completed at least two of three measurement sessions. Groups did not differ according to patients’ characteristics (Table 1) and training compliance (IG 58.8%, 95% CI 48.9–66.7; CG 60.8%, 95% CI 41.2–68; *p* = 0.962). A total of 99.1% of IG’s exercise sessions were performed as prescribed or intensity-reduced, while two sessions had to be stopped prematurely—one because of knee pain and one because of discomfort. In the CG, one session had to be stopped because of discomfort. No severe adverse events (e.g., bleeding, collapse, muscle rupture) occurred. Differences between groups were apparent at baseline in power during the counter-movement jump, where CG performed better than IG. The linear regression model exhibited no VO_2peak_ group difference (CG minus IG) at T1 (estimated as − 0.08 l/min; 95% CI −0.22–0.07). It also revealed that higher age led to lower VO_2peak_ and high VO_2peak_ baseline values led to higher values at hospital discharge, whereas gender did not seem to influence VO_2peak_.

### Cardiorespiratory fitness

**Fig. 2:**
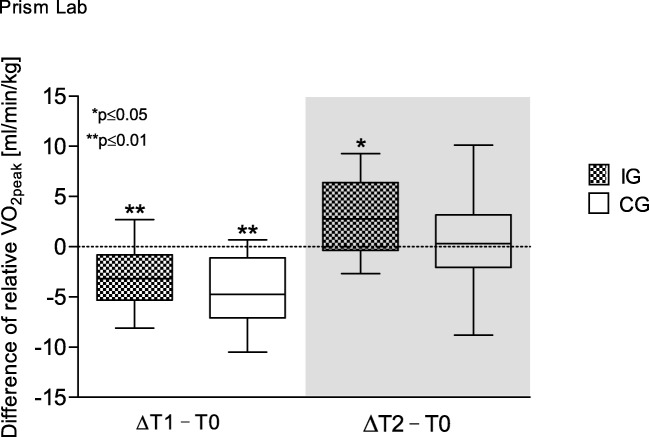
Difference of relative VO_2peak_ over time. Box and whisker plots showing the lower quartile (25th percentile), median (50th percentile), upper quartile (75th percentile), and degree of dispersion as 95% confidence interval (95% CI)

**Fig. 3:**
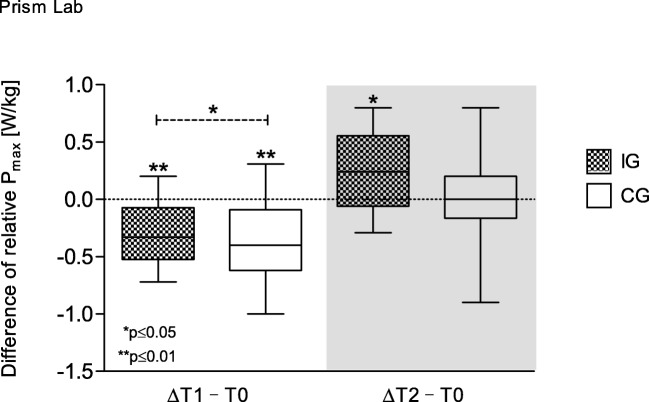
Difference of relative maximum power over time. Box and whisker plots showing the lower quartile (25th percentile), median (50th percentile), upper quartile (75th percentile), and degree of dispersion as 95% confidence interval (95% CI)

The VO_2peak_ decreased significantly during both groups’ hospitalization (IG *p* = 0.002; CG *p* = 0.000) and regained initial values after follow-up. Results of VO_2peak_ and maximum power considering patients’ body weight present differences between groups: during hospitalization both groups showed a decrease of maximum power (IG *p* = 0.005; CG *p* = 0.000) and VO_2peak_ (IG p = 0.002; CG p = 0.000), whereas at follow-up, only IG’s VO_2peak_ (*p* = 0.035) and maximum power (*p* = 0.011) significantly increased (Figs. 2 and 3).

### Strength capacity

**Fig. 4:**
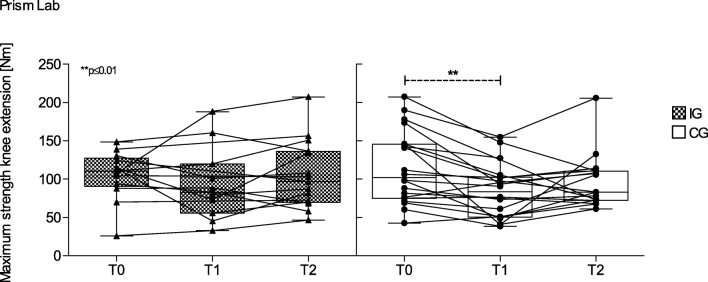
Maximum strength of knee extensors. Box and whisker plots showing the lower quartile (25th percentile), median (50th percentile), upper quartile (75th percentile), and degree of dispersion as 95% confidence interval (95% CI). Dot plots showing individual values of participants of each group

After hospitalization, CG’s maximum strength capacity of the knee extensors and flexors was significantly reduced (extension *p* = 0.003; flexion *p* = 0.044), while IG’s values remained unchanged (Fig. 4). We observed no changes after follow-up. Strength endurance capacity did not change during hospitalization or at follow-up in either group.

### Functional performance

**Fig. 5:**
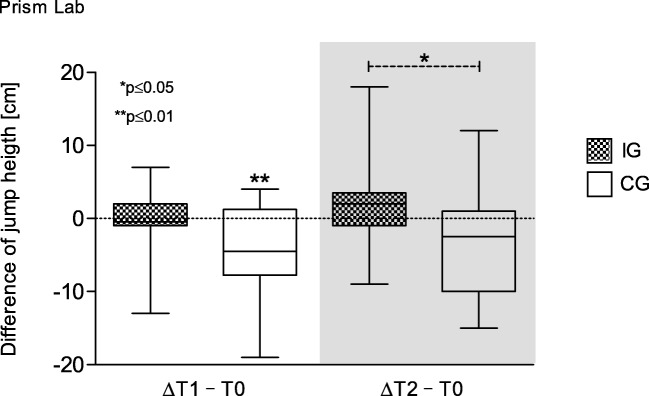
Difference of jump height over time. Box and whisker plots showing the lower quartile (25th percentile), median (50th percentile), upper quartile (75th percentile), and degree of dispersion as 95% confidence interval (95% CI)

CG’s jump height is significantly reduced at hospital discharge (*p* = 0.005). At follow-up, significantly different values between groups in favor of the IG are shown (*p* = 0.033) (Fig. 5). Maximum power during jumping is also only significantly reduced in the CG at T1 (*p* = 0.039). Both groups’ time and power output during the chair-rising test remained unchanged after hospitalization but improved significantly in the IG at follow-up (time *p* = 0.022; power *p* = 0.009).

### Body composition

Many body composition parameters decreased during hospitalization in both groups and exhibited similar changes between groups and the time of measurement, unlike the fat-free mass (%) which remained unchanged in both groups. We noted differences in body cell mass and phase angle after follow-up between groups (Table 2): the IG presented an increase in the fat-free mass (*p* = 0.035), but the CG did not. Furthermore, only the CG’s body cell mass was significantly reduced (*p* = 0.002), while the IG’s regained the baseline level. We noted the same with the phase angle, which regained baseline level at T2, while the CG’s was significantly reduced (*p* = 0.002).

**Table 2:** Results of primary and secondary outcomes

	N		T0 median (range)	N	T1 median (range)	N	T2 median (range)	N	ΔT1–T0 Hodges-Lehman (95% CI)		N	ΔT2–T0 Hodges-Lehman (95% CI)	
Cardiorespiratory fitness													
VO2peak (l/min)	IG	18	1.9 (1.2–2)	14	1.2 (1–1.5)	17	1.8 (1.4–2.3)	16	− 0.5 (− 0.7–− 0.3)	**	17	0.1 (− 0.2–0.2)	
	CG	23	1.7 (1.4–1.9)	23	1.2 (1–1.3)	22	1.5 (1.3–1.8)	22	− 0.5 (− 0.7– − 0.4)	**	20	− 0.2 (− 0.4–0.1)	
VO2peak_rel (ml/min/kg)	IG	18	19.5 (17.4–24.4)	14	16.9 (15–18.6)	17	23.6 (18.9–26.3)	16	− 5 (− 7.5–− 3)	**	17	2.4 (0.3–4.4)	*
	CG	23	25.7 (22.9–27.9)	23	17.9 (15.8–21.5)	22	25 (19.2–27.1)	22	− 6 (− 7.9–− 4.5)		20	− 0.7 (− 2.6–1.9)	
Pmax (W/kg)	IG	18	1.3 (1.1–1.7)	14	1.2 (1–1.3)	17	1.7 (1.3–2.1)	16	− 0.3 (− 0.5–− 0.1)	**	17	0.3 (0.1–0.4)	*
	CG	23	1.7 (1.5–1.9)	23	1.3 (1–1.6)	22	1.7 (1.3–1.9)	22	− 0.4 (− 0.5–− 0.2)	**	20	0 (− 0.2–0.2)	
									*				
Strength capacity													
EXmax (Nm)	IG	13	111 (93.8–124.3)	10	85.8 (64.7–124.4)	15	96.9 (72.7–136.7)	10	− 5.1 (− 35.7–35.1)		13	− 1 (− 19.6–21.3)	
	CG	20	102.4 (78.9–145.9)	18	83.8 (51.2–101.6)	18	83.2 (72.7–109.6)	18	− 24.9 (− 44–− 9.9)	**	16	− 14.1 (− 42.3–4.2)	
FLEXmax (Nm)	IG	13	61 (51.6–66.4)	10	46.1 (35.3–76.6)	15	60.9 (56.2–92.4)	10	− 5.2 (− 17.3–10.2)		13	8.9 (− 5–21.6)	
	CG	20	64.3 (52.2–88.6)	18	53 (35.1–60.7)	18	61.8 (46.6–70.6)	18	− 6.1 (− 22.9–− 0.7)	*	16	− 3.8 (− 19.5–7.7)	
EXendurance (%)	IG	13	82.3 (75.2–96)	10	84.3 (74–89.6)	15	83.5 (74.6–95.3)	10	− 4.4 (− 33.2–9.1)		13	0.2 (− 29.6–10.6)	
	CG	20	81.7 (73.4–90.7)	18	82.9 (76.1–86.5)	18	78.5 (71.2–88.9)	18	− 0.5 (− 9–5.4)		16	− 5.1 (− 16.8–3.6)	
FLEXendurance (%)	IG	13	84.8 (77.3–100.2)	10	94.4 (85–99.1)	15	84.3 (80.1–107.8)	10	0.7 (− 14.5–14.4)		13	2.3 (− 10.3–10.5)	
	CG	20	88.7 (82.3–96.6)	18	85.3 (74.8–89.6)	18	82.7 (75.3–88.8)	18	− 4.2 (− 14.8–5.6)		16	− 8.3 (− 19.4–1.7)	
Functional performance													
HeightCMJ (cm)	IG	16	23.8 (16.7–26.4)	12	22.2 (19.4–28.7)	14	29.7 (24.3–30.4)	14	−0 (−5.1–3.1)		13	2.8 (−1.4–6.2)	
	CG	23	30.4 (22.4–36.4)	16	22.8 (19.5–31.5)	21	29.4 (19.1–31.9)	17	−5.4 (−10 – −1.8)	**	20	−2.7 (−6.8–0.8)	
												*	
PCMJ (W/kg)	IG	16	26.4 (20.3–29.6)	12	25.2 (24.5–28.5)	14	30.7 (25.5–32.1)	14	− 1.9 (− 4.9–1.2)		13	2.1 (− 0.7–4.2)	
	CG	23	30.6 (27.5–36.2)	16	27 (19.5–31.5)	21	31.1 (24.9–36.7)	17	− 4.6 (− 9.8–− 0.2)	*	20	− 1.7 (− 5.4–1.8)	
			*										
TimeCRT (s)	IG	17	2.4 (2.2–2.8)	13	2.2 (2–2.7)	15	1.8 (1.7–2.1)	15	− 0 (− 0.2–0.3)		14	− 0.4 (− 0.6–− 0.1)	*
	CG	22	1.9 (1.8–2.2)	15	2.5 (2–3.1)	20	2.1 (1.7–2.6)	16	0.3 (− 0.2–1.4)		18	− 0.1 (− 0.4–0.5)	
PCRT (W/kg)	IG	17	9.8 (7.4–11.6)	13	8.8 (8.5–11.7)	15	12.1 (9.5–14.4)	15	− 0.2 (− 1.4–0.9)		14	2 (0.9–3.7)	**
	CG	22	10.4 (8.9–13.1)	15	10 (5.5–10.6)	20	12.2 (8.7–14.9)	16	− 1.8 (− 4–0.2)		18	1.4 (− 0.8–3)	
Body composition													
BMI (kg/m2)	IG	18	26.1 (23.8–31.4)	18	24.2 (22.5–28.3)	18	25.3 (21.2–26.4)	18	− 2 (− 2.5–− 1.5)	**	18	− 2 (− 2.9–− 1.1)	**
	CG	26	24.9 (23.5–26.3)	25	22.7 (20.5–24.3)	22	21.8 (20.5–23.8)	25	− 1.6 (− 2–− 1.2)	**	22	− 2.5 (− 3.8–− 1.4)	**
FMBODPOD (%)	IG	18	37 (33–43.2)	16	37.7 (31.6–43)	18	32.5 (24.1–41.8)	16	− 0.2 (− 1–0.7)		18	− 4 (− 6.4–− 1.9)	**
	CG	25	32.5 (29.5–36.3)	24	33.8 (32.5–36.5)	22	29.1 (22.5–31.8)	23	− 0.1 (− 1.2–1.1)		22	− 4.8 (− 7–− 2.5)	**
FMBIA (kg)	IG	18	22.7 (17.4–28.2)	18	17.2 (13.6–24.3)	18	15.7 (11.1–23.4)	18	− 3 (− 4.1–− 2.2)	**	18	− 6.1 (− 9.4–− 2.6)	**
	CG	25	17.7 (14.4–22.8)	25	15.1 (10.2–18.1)	21	11 (8–13.9)	24	− 2.3 (− 5.5–− 0.8)	**	23	− 6 (− 9.9–− 3.4)	**
FFMBODPOD (%)	IG	18	63 (56.9–67)	16	62.3 (57–68.4)	18	65.6 (56.2–73.1)	16	0.2 (− 0.7–1)		18	3.4 (0.4–5.6)	*
	CG	25	65.8 (63–70)	24	65.7 (63.2–67.4)	22	69.3 (64.8–75.7)	23	0.2 (− 1–1.4)		22	4.3 (− 1.3–7.7)	
BCM (kg)	IG	18	29.3 (22.1–33.3)	18	25.4 (19.9–29.8)	18	25.8 (22.4–32)	18	− 3 (− 4.4–− 1.8)	**	18	− 1.3 (− 3–0.3)	
	CG	25	28.1 (24.1–32.4)	25	24.1 (20.9–27.2)	21	24.9 (21.1–27.9)	24	− 3.4 (− 4.5–− 2.4)	**	23	− 4 (− 5.7–− 2)	**
Phase angle (°)	IG	18	5.3 (4.8–5.5)	18	4.8 (4.3–5.2)	18	5 (4.5–5.5)	18	− 0.5 (− 0.7–− 0.2)	**	18	− 0.3 (− 0.6–0.1)	
	CG	25	5.2 (5–6.1)	25	4.7 (4.3–5.2)	21	4.7 (4.2–4.7)	24	− 0.7 (− 1.1–− 0.4)	**	22	− 0.9 (− 1.4–− 0.4)	**
Quality of life EORTC QLQ-C30													
Global QoL (%)	IG	17	58.3 (50–62.5)	16	41.7 (33.3–58.3)	18	70.8 (66.7–83.3)	15	− 8.3 (− 20.8–12.5)		17	16.7 (8.3–29.2)	*
	CG	23	66.7 (50–75)	25	41.7 (33.3–50)	24	75 (58.3–83.3)	23	− 12.5 (− 25–− 4.2)	*	22	8.3 (0–20.8)	*
Physical functioning (%)	IG	17	80 (60–86.7)	16	73.3 (53.3–80)	18	86.7 (80–96.7)	15	− 7.5 (− 20–5.8)		17	6.7 (0–16.7)	*
	CG	23	80 (73.3–93.3)	25	60 (46.7–66.7)	24	73.3 (60–86.7)	25	− 20 (− 30–− 10)	**	24	− 1.7 (− 10–6.7)	
Role functioning (%)	IG	17	50 (33.3–66.7)	16	25 (0–33.3)	18	66.7 (58.3–100)	15	− 16.7(− 50–16.7)		17	25 (0–50)	*
	CG	23	50 (16.7–75)	23	33.3 (0–33.3)	24	66.7 (33.3–66.7)	22	− 25(− 41.7–8.3)		23	8.3 (− 8.3–33.3)	
Emotional functioning (%)	IG	17	58.3 (50–75)	16	66.7 (50–75)	18	79.2 (75–91.7)	15	8.3(− 8.3–16.7)		17	16.7 (8.3–29.2)	**
	CG	23	75 (66.7–83.3)	23	66.7 (66.7–83.3)	24	66.7 (58.3–83.3)	24	− 8.3(− 16.7–0)		23	− 4.2 (− 16.7–8.3)	
												**	
Cognitive functioning (%)	IG	17	66.7 (58.3–83.3)	16	66.7 (80–83.3)	18	75 (66.7–83.3)	15	0 (− 16.7–8.3)		17	8.3 (− 8.3–16.7)	
	CG	23	83.3 (66.7–100)	23	83.3 (66.7–83.3)	24	83.3 (66.7–100)	24	−8.3 (−8.3–0)		23	0 (−8.3–8.3)	
Social functioning (%)	IG	17	50 (16.7–66.7)	16	33.3 (16.7–50)	18	66.7 (58.3–66.7)	15	− 8.3 (− 33.3–16.7)		17	16.7 (8.2–33.3)	*
	CG	23	66.7 (50–66.7)	23	50 (16.7–66.7)	24	66.7 (50–74.8)	23	− 8.3 (− 16.7–0)		23	8.3 (− 8.3–16.7)	
EORTC QLQ-HDC29													
Gastro-intestinal side effects (%)	IG	17	13.3 (0–26.7)	16	33.3 (26.7–46.7)	17	20 (6.7–26.7)		20 (10–30)	**		3.3 (− 3.3–6.7)	
	CG	25	6.7 (0–13.3)	24	33.3 (13.3–40)	24	23.3 (13.3–40)		23.3 (10–33.3)	**		13.3 (3.3–26.7)	*
Skin problems (score)	IG	16	0 (0 33.3)	16	16.7 (0–33.3)	16	33.3 (33.3–33.3)		0 (− 33.3–16.7)			16.7 (− 16.7–33.3)	
	CG	24	0 (0–33.3)	24	33.3 (0–33.3)	24	50 (33.3–66.7)		16.7 (16.7–33.3)	**		33.3 (16.7–50)	*
Fever or infections (score)	IG	16	0 (0–33.3)	16	0 (0–66.7)	16	0 (0–33.3)		0 (− 33.3–33.3)			0 (0–33.3)	
	CG	24	0 (0–0)	24	0 (0–33.3)	24	0 (0–0)		0 (− 16.7–16.7)			0 (− 16.7–0)	
Aches in bones (score)	IG	16	0 (0–33.3)	16	33.3 (0–33.3)	16	0 (0–0)		16.7 (− 16.7–50)			0 (− 16.7–33.3)	
	CG	24	0 (0–33.3)	24	0 (0–33.3)	24	0 (0–33.3)		0 (0–16.7)			0 (0–0)	
Urine frequency (score)	IG	16	33.3 (0–66.7)	16	50 (33.3–100)	16	0 (0–33.3)		33.3 (− 16.7–66.7)			0 (− 33.3–16.7)	
	CG	24	16.7 (0–33.3)	24	33.3 (0–66.7)	24	0 (0–33.3)		0 (− 16.7–16.7)			0 (− 16.7–0)	
									*				
Multidimensional fatigue inventory questionnaire (MFI)													
MFI (total score)	IG	17	63 (41–68)	16	60.5 (50–67)	18	41.5 (29.5–51)	15	3 (− 11–17)		17	− 12.5 (− 21.5– −5.5)	**
	CG	25	52 (41–58.5)	25	62 (56–71)	24	50 (37–61)	25	10 (3.5–17)	**	24	− 1 (− 10.5–7.5)	
												*	

*p ≤ 0.05

**p ≤ 0.01

*Pmax*, maximum power output; *EX*, strength of the knee extensors; *FLEX*, strength of the knee flexors; *PCMJ*, power output during counter-movement jump; *PCRT*, power output during chair-rising test; *FM*, fat mass; *FFM*, fat-free mass; *BCM*, body cell mass; *BIA*, bioelectrical impendance analysis; *BODPOD*, Bod Pod Body; *QoL*, quality of life

### Quality of life and fatigue

After hospitalization, CG patients reported reduced QoL (*p* = 0.015), while the IG’s QoL remained unchanged. After follow-up, both groups reported significantly better QoL (IG *p* = 0.013; CG *p* = 0.037). The CG’s physical functioning worsened during hospitalization (*p* = 0.001). In contrast, IG reported improved physical functioning at follow-up (*p* = 0.035), as well as improved role functioning (*p* = 0.025), emotional functioning (p = 0.002), and social functioning (*p* = 0.014). Furthermore, both groups reported worsening gastrointestinal side effects (IG p = 0.001; CG p = 0.001) after hospitalization. The CG also reported worse gastrointestinal side effects 6 months after transplantation than at baseline (*p* = 0.019). Exacerbated skin problems were only reported by the CG after hospitalization (*p* = 0.001) and 6 months after transplantation (*p* = 0.026). Urine frequency differed between groups after hospitalization (*p* = 0.041).

Furthermore, the CG reported a significantly higher fatigue level at T1 (*p* = 0.005), while the IG’s remained unchanged. They also reported significantly lower fatigue at T2 (*p* = 0.006), while CG’s fatigue returned to baseline level, leading to a significant group difference over time at follow-up (*p* = 0.038).

### Physical activity

The amount of physical activity carried out during sports increased with the initial cancer treatment in both groups (IG *p* = 0.006; CG *p* = 0.000). In contrast, IG reported significantly reduced physical activity during daily routine (*p* = 0.043) and leisure time (*p* = 0.023) after the initial cancer treatment compared with the time before. The CG reduced only their habits regarding leisure time activity (*p* = 0.005) during that time. After alloHCT, both groups reported significantly less physical activity during daily routine (IG *p* = 0.002; CG *p* = 0.000) while the CG also significantly reduced their physical activity during leisure time (*p* = 0.009). We observed no group differences at any time of measurement (Table 3).

**Table 3:** The amount of physical activity during daily routine, leisure time and sports at different points in time.

		N	Before diagnosis median (range)	N	Since cancer therapy median (range)	N	Day ± 180 after alloHCT median (range)	Δ^a^ Hodges-Lehman (95% CI)		Δ^b^ Hodges-Lehman (95% CI)	
Amount of physical activity											
Daily routine (kJ)	IG	18	28,460.6 (16,773.3–41,152.6)	18	28,012.7 (18,091.9–39,829.8)	18	22,470.4 (14,638.4–31,382.4)	0 (−460.5–0)	*	− 2520 (− 4374.4–− 1209.8)	**
	CG	25	21,111 (15191–26,191.8)	24	21,997.4 (16,149.6–32,345.2)	22	16,526.3 (13,072.9–21,624.9)	0 (0–0)		− 2402.8 (− 4357.6–− 1218.1)	**
Leisure time (kJ)	IG	17	7467.8 (2566–11,344)	17	1080 (0–5554.8)	18	3641.8 (925.1–7438.5)	− 4386.9 (− 6852.5–− 1469.3)	*	− 3750.7 (− 6865–100.5)	
	CG	26	7949.2 (2980.4–11,766.8)	26	2804.6 (1335.3–5847.8)	22	2524.2 (1511.2–5927.4)	− 2888.3 (− 5776.7–− 46.1)	**	− 5027.4 (− 9837.1–− 996.3)	**
Sports (kJ)	IG	15	0 (0–1322.8)	15	26,853.2 (0–61,057)	12	0 (0–1565.6)	31,562.4 (4508.3–76,821.5)	**	0 (− 1410.7–2247.9)	
	CG	23	0 (0–2570.2)	25	21,968.1 (4922.7–50,751.1)	17	0 (0–5617.6)	28,962.9 (10,984.1–45,309.3)	**	0 (− 7350.6–2867.4)	

*p < 0.05

**p < 0.01

^a^Difference between the time before cancer treatment and the time since cancer treatment

^b^Difference between the time before cancer treatment and the time after alloHCT

## Discussion

To best of our knowledge, no study so far had investigated the effects of WBV during alloHCT and we are the first to have implemented a CPET immediately upon hospital discharge. The feasibility of both can be confirmed, as we detected no adverse events during training or testing and our dropout rate is comparable to other interventional studies [15, 19, 46].

By implementing WBV during alloHCT, we observed a clear benefit in cardiorespiratory fitness in our IG in contrast to the CG 6 months after transplantation. We suppose that CPET performance at hospital discharge was considerably influenced by medical treatment and treatment-related side effects that impaired patients’ ability to realize their maximum capacity and that obliterated the differences between groups at hospital discharge. We hypothesized that otherwise, their maximum oxygen uptake could have been maintained by WBV, as it is rose during acute exercising [47]. Increased metabolic power due to enhanced muscular activity and a higher heart rate and lactate concentration similar to aerobic endurance exercises such as during WBV are factors supporting this hypothesis [39, 48]. Mester et al. [40] assumed that more efficient gas exchange and material metabolism between blood and muscle fibers (thanks to more opened capillaries immediately after WBV) could be one reason for increased oxygen uptake after exercise. In line with this, a WBV study in patients with pulmonary arterial hypertension revealed a VO_2peak_ increase after only 4 weeks’ hospitalization [33]. Their exercise prescription resembled our study. However, as our patients’ diagnosis and subsequent therapy differed from the aforementioned study, we assume the medical treatment during hospitalization is the main reason for our cardiorespiratory fitness findings at hospital discharge. Nevertheless, we assume that WBV during hospitalization plays a crucial role in ensuring the IG’s better cardiorespiratory fitness 6 months after transplantation. First of all, patients in both groups underwent similar physical conditioning before alloHCT—objectively measured via HCT-CI scores, the Karnofsky performance index and our baseline assessments, and subjectively, the EORTC-HDC29-questionnaire. We detected no differences between groups at baseline through our assessments and baseline characteristics, thus we assume that no group was in better physical condition before transplantation. Furthermore, both groups’ hospitalization times were the same, which led us to assume that both groups’ medical treatment routines were also similar and that they had to handle the same transplant-associated side effects. Although patients were not monitored from hospital discharge till follow-up, the reported amount of physical activity during that time period was similar between groups. This fact had led us to assume that WBV did indeed affect VO_2peak_ and power, quantifiable only after follow-up. As cardiorespiratory fitness is defined by interaction between muscles, the heart, and lungs [23], muscle strength can be regarded as a major criterion of those systems’ functioning. Thus almost maintaining the strength of knee extensors and flexors during hospitalization may have facilitated the cardiorespiratory system’s ability to recover faster after hospital discharge. Furthermore, WBV might have induced cardiovascular orthostatic stress that reduces the risk of cardiovascular deconditioning caused by bed rest [49, 50]. There are indications that exercising in upright position can accelerate the rehabilitation process due to increased orthostatic stress, unlike exercising in supine position [51, 52] which has mostly been carried out in the CG. Additionally, WBV increases blood flow [53] that may also encourage cardiovascular adaptations.

The aforementioned accelerated physical recovery of the IG compared with the CG is also reflected by enhanced cell metabolism. The IG’s body cell mass, which consists of all metabolically active and protein-rich intracellular tissue inter alia muscle mass [54], can be a predictor of malnutrition [55]. It regained baseline values at follow-up, while the CG’s dropped. The phase angle also indicates improved metabolism; by measuring cellular membrane integrity and alterations of fluid balance [56, 57], the phase angle provides information about the nutritional status of patients and may predict their clinical prognosis [58]. A phase angle improvement could lead to enhanced recovery of the muscular [59] and cardiorespiratory system, and may thus result in a better overall survival [57].

Our findings concerning maximum leg strength and functional performance are in line with other working groups who observed improved strength capacity [60, 61] or improved functional performance, i.e., jump height after WBV, especially in weak or untrained persons [62, 63]. Despite the low amount of IG’s maximum leg strength data due to interim technical problems and absolute values which could not be maintained completely, a difference towards the CG which significantly loses maximum leg strength is shown. Fitts et al. and Widrick et al. [64, 65] observed reduced neuronal activation especially during the first days to weeks of immobilization as a key reason for reduced maximum strength and power afterwards. As WBV enhances the recruitment of motor units [30, 31], we assume that WBV can reduce the effects of bed rest on muscle strength by maintaining inter- and intramuscular coordination. Furthermore, WBV influences the stretch-shortening cycle involved significantly in jumping performance [66, 67]. We thus suspect that WBV-induced neuro-muscular stimulation inhibited the loss of strength of the legs and functional performance during hospitalization [68]. But since the IG’s muscle strength and jumping performance did not improve further until follow-up, we assume that WBV’s benefits vanished because of discontinued exercising. In contrast, the IG’s performance during the chair-rising test improved at follow-up. We assume that this improvement is more likely due to everyday practice at home—less likely a long-term effect of WBV.

Our results regarding fatigue are comparable to other studies that observed less fatigue due to physical activity during hospitalization in cancer patients [69, 70]. Wiskemann et al. [19] demonstrated that psychological symptoms correlate inversely with physiological performance. Furthermore, and in line with our results, they demonstrated reduced fatigue levels 6 to 8 weeks after hospital discharge. There is evidence that in addition to medical treatment, the fatigue level also depends on physiological factors, such as the hemoglobin level and physical performance [71–73]. Since both groups received the same amount of social contact, we imagine that the IG’s aforementioned better physical condition strengthened patients’ individual psychological resources and thus led to differences in fatigue; QoL; and reported physical, emotional, social, and role functioning between groups at both hospital discharge and follow-up.

Taking together, WBV seems to affect the cardiorespiratory, neuromuscular, and intracellular systems during alloHCT in different ways: strength of the knee extensors and flexors and jumping performance are directly influenced by enhancing muscular coordination and may be described as acute WBV-triggered adaptations during alloHCT. Cardiorespiratory fitness in turn is indirectly affected and quantifiable only in the long-term, as are the phase angle and body cell mass. We assume that these divergences mainly represent the physiological mechanism of WBV and different abilities of cardiorespiratory, neuromuscular, and intracellular systems to recover after alloHCT. Importantly, WBV seemed to improve psychosocial factors even during hospitalization, persisting for at least 6 months after transplantation.

Our study did not investigate the relative benefit of WBV against conventional resistance training during alloHCT, which is why we are unable to attribute our results to WBV exercises explicitly. We instead aimed to introduce a promising exercise method that may directly and indirectly affect as many physiological and psychosocial aspects as possible relevant for alloHCT patients. Although we have described an effective intervention, we propose comparing different types of exercise with greater sample sizes in future studies to reveal best practice. Furthermore, it might be beneficial to continue intervention exercises long-term after alloHCT to ensure successful overall rehabilitation.

## Conclusion

WBV presents an effective exercise method for patients undergoing alloHCT to preserve maximum strength of leg muscles, functional performance, and QoL, as well as to prevent worsening fatigue during hospitalization. Furthermore, WBV seems to facilitate accelerated physical recovery concerning the cardiorespiratory system, body cell mass, and phase angle. Our IG’s superior physical conditioning after follow-up may have entailed better QoL and reduced fatigue level. We conclude that our intervention succeeded by enabling a treatment strategy that improves alloHCT patients’ physical and psychological well-being. However, to define best practice, we propose to expand the pre- and post-alloHCT intervention period, to investigate the relative benefit of different types of exercises on physiological parameters, and to evaluate their impact on survival.

## Electronic supplementary material

ESM 1 (DOCX 26 kb)

ESM 2 (DOCX 23 kb)

## Data Availability

The dataset generated and analyzed during the current study are available from the corresponding author on reasonable request.
