# Supplementary material for: Whole body vibration training during allogeneic hematopoietic cell transplantation—the effects on patients’ physical capacity
Source: Ann Hematol. 2020 Jan 23;99(3):635–48. doi: 10.1007/s00277-020-03921-x (PMC7060160; doi:10.1007/s00277-020-03921-x)
Supplement: Supplementary file 1 — (DOCX 26 kb) [file 277_2020_3921_MOESM1_ESM.docx]

Title

Whole body vibration training during allogeneic hematopoietic cell transplantation – The effects on patients' physical capacity

Authors

Antonia Pahl^1#^, Anja Wehrle^2^, Sarah Kneis^1^, Albert Gollhofer^3^, Hartmut Bertz^1^

Affiliations

^1^Department of Medicine I, Medical Center – University of Freiburg, Faculty of Medicine, Hugstetterstr. 55, 79106 Freiburg, Germany

^2^Institute for Exercise- and Occupational Medicine, Medical Center – University of Freiburg, Faculty of Medicine, Hugstetterstr. 55, 79106 Freiburg, Germany

^3^Department of Sport and Sport Science, University of Freiburg, Schwarzwaldstraße 175, 79117 Freiburg, Germany

^#^Corresponding author

antonia.pahl@uniklinik-freiburg.de

# Supplementary file 1 Methods

## Intervention

We controlled patients' blood pressure before and after, and their heart rate before, during, and after exercising to avoid overload. Blood values, blood pressure, heart rate, training progress, and reasons for missed exercise sessions as well as any extra physical exercises during hospitalization were documented.
Our WBV training protocol included at least five different exercises from a repertoire of 16 exercises for training the lower limbs, especially the knee extensors and flexors as in previous investigations [1–3]. WBV's intensity was determined by the vibration frequency (Hz) and peak-to-peak displacement. The latter we defined by the amplitude, which was determined by the second toe’s position on the vibration platform, multiplied by two [4]. Exercises were graded by the level of difficulty and exertion (Supplementary file 2) and were done static- or dynamically barefoot on the vibration platform. During static exercises, patients were asked to shift their body weight onto their forefeet and to maintain a knee angle of approx. 60 degrees flexion [5]. Speed of movement during dynamic exercises was determined individually by each patient's perception. Due to the infection risk, we disinfected the vibration platform before and after each exercise session, and the floor around the vibration platform was covered with a towel. Each exercise lasted 2min with 1–2min rest between exercises. Patients were to achieve a net exercise time lasting at least 10min and an intensity goal of 14 to 16 on the perceived exertion rating scale [6]. The control group (CG) was to attain a low intensity prescription of 9–11. While WBV was always done in standing position, CG's patients did not always get out of bed unless they explicitly wanted to for their mobilization and stretching exercises

## Cardiorespiratory fitness and peak oxygen consumption

Patients performed an incremental cardiorespiratory exercise test on an electronically braked cycle ergometer (ergoline ergoselect 1200, Ergoline GmbH, Bitz, Germany) in recumbent (40°) position during continuous monitoring of their ECG, heart rate, and blood pressure. Gas exchange and ventilation were recorded continuously via breath-by-breath gas analysis (MetaLyzer 3B-R3, Cortex Biophysik GmbH, Leipzip, Germany). The patients had to perform until total exhaustion starting at 20 Watt with a 10-Watt increase every minute.

## Strength capacity

The strength test was limited to the knee-extensor and flexor muscles, as their capacity determines patients' maximum power during cardiorespiratory exercise testing; the WBV exercise sessions focused on training those muscles. To become familiar with the test procedure, patients did eight submaximal repetitions of our test protocol, whose maximum voluntary strength amounted to five maximum concentric contractions at 60 degrees/second, whereas the muscular-endurance protocol called for 20 maximum concentric contractions at 180 degrees/second. We used the highest value for extension and flexion at 60 degrees/second to analyze maximum capacity. Muscular endurance was defined as the percentage loss of strength over all 20 repetitions at 180 degrees/second, which was calculated as: $Endurance (\%)=\frac{\emptyset repetition 16 -20}{\left( \emptyset repetition 2 -6 \right)*100}$

## Functional performance

The force plate determined dynamic ground reaction forces in their local and temporal progress. Data were recorded with a sample rate of 800Hz and analyzed using Leonardo Mechanograph® Research-Software (Novotec Medical GmbH, Pforzheim, Germany).

For CRT, patients started in a sitting position with their arms lying across their chest. Patients had to stand up from a bench (45cm, Novotec Medical GmbH) and sit down again five times in a row. They were asked to rise until their knees and hips were fully extended, and to sit down using their full bodyweight. The duration of one repetition was calculated averaging the second, third, and fourth repetitions. For CMJ, patients were told to jump as high as possible moving their arms freely.

## Quality of Life and Fatigue

The EORTC QLQ-C30-questionnaire includes global quality of life and five different functional scales: physical functioning, role functioning, emotional functioning, cognitive functioning, and social functioning. Scores were scaled from 0% to 100% with higher scores indicating better QoL [7]. The EORTC-HDC29-questionnaire includes different scales and single items covering physical and psychosocial symptoms typically associated with stem cell transplantation; its scale is identical to the EORTC-QLQ-C30-questionnaire’s. We only applied the physical symptom scales and items for analysis. The Multidimensional Fatigue Inventory questionnaire is a 20-item, self-assess instrument and covers different dimensions of fatigue [8]; each question has a 1–to5–point scale, we calculated a total score on all questions. Higher score (maximum 100 for total score) indicates greater fatigue.

## Physical activity

Calories lost during physical activity were calculated by relying on the metabolic equivalent of each type of exercise [9].

## References

1. Álvarez-Barbosa F, del Pozo-Cruz J, del Pozo-Cruz B, Alfonso-Rosa RM, Rogers ME, Zhang Y. Effects of supervised whole body vibration exercise on fall risk factors, functional dependence and health-related quality of life in nursing home residents aged 80+. Maturitas. 2014;79:456–63.

2. Delecluse C, Roelants M, Verschueren S. Strength Increase after Whole-Body Vibration Compared with Resistance Training. Med Sci Sports Exerc. 2003;35. http://journals.lww.com/acsm-msse/Fulltext/2003/06000/Strength_Increase_after_Whole_Body_Vibration.21.aspx.

3. Liao L-R, Ng GYF, Jones AYM, Huang M-Z, Pang MYC. Whole-Body Vibration Intensities in Chronic Stroke: A Randomized Controlled Trial. Med Sci Sports Exerc. 2016.

4. Rauch F, Sievanen H, Boonen S, Cardinale M, Degens H, Felsenberg D, et al. Reporting whole-body vibration intervention studies: recommendations of the International Society of Musculoskeletal and Neuronal Interactions. J Musculoskelet Neuronal Interact. 2010;10:193–8.

5. Ritzmann R, Gollhofer A, Kramer A. The influence of vibration type, frequency, body position and additional load on the neuromuscular activity during whole body vibration. Eur J Appl Physiol. 2013;113:1–11.

6. Borg G. Psychophysical bases of perceived exertion. Med Sci Sports Exerc. 1982;14:377–81.

7. Fayers PM, Aaronson N, Bjordal K, Groenvold M, Curran D, Bottomley A, et al. EORTC QLQ-C30 scoring manual: this manual is intended to assist users with scoring procedures for the QLQ-C30 version 3 and earlier, and the QLQ supplementary modules. Brussels: EORTC; 2001.

8. Smets EM, Garssen B, Bonke B, De Haes JC. The Multidimensional Fatigue Inventory (MFI) psychometric qualities of an instrument to assess fatigue. J Psychosom Res. 1995;39:315–25.

9. Frey I, Berg A, Grathwohl D, Keul J. Freiburger Fragebogen zur körperlichen Aktivität-Entwicklung, Prüfung und Anwendung. Soz- Präventivmedizin. 1999;44:55–64.
