# Supplementary material for: Whole body vibration training during allogeneic hematopoietic cell transplantation—the effects on patients’ physical capacity
Source: Ann Hematol. 2020 Jan 23;99(3):635–48. doi: 10.1007/s00277-020-03921-x (PMC7060160; doi:10.1007/s00277-020-03921-x)
Supplement: Supplementary file 2 — (DOCX 23 kb) [file 277_2020_3921_MOESM2_ESM.docx]

Title

Whole body vibration training during allogeneic hematopoietic cell transplantation – The effects on patients' physical capacity

Authors

Antonia Pahl^1#^, Anja Wehrle^2^, Sarah Kneis^1^, Albert Gollhofer^3^, Hartmut Bertz^1^

Affiliations

^1^Department of Medicine I, Medical Center – University of Freiburg, Faculty of Medicine, Hugstetterstr. 55, 79106 Freiburg, Germany

^2^Institute for Exercise- and Occupational Medicine, Medical Center – University of Freiburg, Faculty of Medicine, Hugstetterstr. 55, 79106 Freiburg, Germany

^3^Department of Sport and Sport Science, University of Freiburg, Schwarzwaldstraße 175, 79117 Freiburg, Germany

^#^Corresponding author

antonia.pahl@uniklinik-freiburg.de

**Supplementary file 2** Exercise repertoire of whole body vibration training

| **Number** | **Level of difficulty** | **Exercise** | **Intensity** | | | | |
| --- | --- | --- | --- | --- | --- | --- | --- |
|  |  |  | Level I  20-22,5Hz | | Level II  23-24,5Hz | | Level III  25-27 Hz |
| 1 | Easy | **Upright standing**  *Amplitude* 2 |  | |  | |  |
| 2 | Easy | **Heel raise on Airex**  *Amplitude* 1,5 |  | |  | |  |
| 3 | Easy | **Tilt hip**  *Amplitude* 2 |  | |  | |  |
| 4 | Easy | **Slight squat on Airex**  *Amplitude* 2,5 |  | |  | |  |
| 5 | Easy/ moderate | **Climber**  Variation: Biceps Curls with arms  *Amplitude* 1 |  | |  | |  |
| 6 | Easy/ moderate | **Lateral Climber**  Variation: Biceps Curls with arms  *Amplitude:* 1 |  |  | |  | |
| 7 | Moderate | **Back extension**  *Amplitude* 2 |  |  | |  | |
| 8 | Moderate | **Abductors**  *Amplitude* 0-1 |  |  | |  | |
| 9 | Moderate/ intensive | **Squat**  *Amplitude* 2 |  |  | |  | |
| 10 | Moderate/ intensive | **Lunges**  *Amplitude* 0-1 |  |  | |  | |
| 11 | Intensive | **Lunges lateral**  *Amplitude* 2 |  |  | |  | |
| 12 | Moderate | **Heel raise**  *Amplitude* 1,5 |  |  | |  | |
| 13 | Intensive | **Combination: Heel raise and squat**  *Amplitude* 2-3 |  |  | |  | |
| 14 | Intensive | **Squat and arms up**  *Amplitude* 2 |  |  | |  | |
| 15 | Intensive | **Monopedal stand: Hip extension and flexion**  *Amplitude* 0-1 |  |  | |  | |
| 16 | Intensive | **Squat and latissimus exercises**  *Amplitude* 2 |  |  | |  | |
